# Medical practitioners’ experiences and considerations when managing sleep medication for adolescents and young adults: A qualitative study on general practitioners’ and child and adolescent psychiatrists’ reflections on their prescription and deprescription practices of sleep medication for 13–24-year-olds

**DOI:** 10.1080/02813432.2024.2407877

**Published:** 2024-09-30

**Authors:** Nanna Maria Andersen, Ásthildur Árnadóttir, Tora Grauers Willadsen, Gritt Overbeck

**Affiliations:** a Department of Public Health, Center for General Practice, University of Copenhagen, Copenhagen, Denmark; b Research Unit for General Practice, Region Zealand, Denmark

**Keywords:** Adolescents, sleep, melatonin, benzodiazepines, deprescriptions

## Abstract

**Introduction:**

The prevalence of sleep disorders and use of sleep medication, particularly melatonin, are rising among adolescents and young adults (13–24 years). In Denmark, melatonin is approved for use in children with autism and ADHD up to 18 years of age, with other prescriptions being off-label in these age groups. The perspectives of medical practitioners on prescribing sleep medications to this age group remain largely unexplored.

**Aim:**

This study aims to investigate the considerations of general practitioners (GPs) and child and adolescent psychiatrists (psychiatrists) when prescribing and deprescribing sleep medications for 13–24-year-olds.

**Methods:**

We conducted qualitative semi-structured interviews with 10 GPs and six psychiatrists. Data were analyzed using an inductive approach.

**Results:**

Psychiatrists typically prescribed melatonin with the expectation that deprescription would occur in general practice. Despite the universal goal of deprescription, it was hindered by various challenges. GPs identified patient motivation and a clear focus on deprescription as facilitative factors and expressed a need for enhanced emphasis on these aspects in general practice.

**Discussion and implications:**

The findings align with existing prescription trends and literature on factors that promote and inhibit deprescription. The study underscores the complexities of deprescribing sleep medications for adolescents and young adults, suggesting the need for expanded guidelines and enhanced continuing education for GPs.

**Conclusions:**

The research highlights significant discrepancies among medical practitioners regarding the deprescription process of sleep medications for young individuals, complicated by multiple factors. This underscores the need for better guidelines and further studies.

## Introduction

Adequate sleep for adolescents (13–17 years old) and young adults (18–24 years old) is an important factor for both mental and physiological health [1,2], with the recommended sleep duration for adolescents and young adults being 8–10 and 7–9 h per 24 h, respectively [3]. The consequences of inadequate sleep can be manifold, causing both cognitive, attentional, emotional and behavioral difficulties [1,4–6], associated with a decline in academic performance, psychosocial health and somatic health [1,6,7].

Sleep disorders in adolescents and young adults are a complex series of issues ranging from medical disorders such as obstructive sleep apnea, insomnia and circadian rhythm disorders to non-medical factors such as poor sleep hygiene or psychological reasons, with insomnia being the most frequent [8–10]. Sleep disorders are relatively common in these age groups [11–13]. A survey from 2019 among Danish high school students aged 15–25 found that 28% of females and 21% of males have self-reported sleep issues more than once weekly [14].

Mental health has long been associated with sleep disorders [6,15], often presumed to have a bidirectional causality, as inadequate sleep can worsen psychiatric symptoms and vice versa [11]. Sleep disorders are a common comorbidity to ADHD and autism spectrum disorders [16,17]. In recent years, psychiatric diagnoses for adolescents and young adults have been increasing, with conditions such as ADHD, anxiety, depression and eating disorders tripling in Denmark since 2006 [18], and autism diagnoses doubling since 2008 [19]. Concurrently, a noticeable decline of mental wellbeing has been reported among Danish youth in general [20].

For both young adults and adolescents with or without an ADHD or autism diagnosis, the Danish Health Authorities recommend non-pharmacological interventions, such as improving sleep hygiene, before intervening with sleep medication [21–24]. The prescription of conventional sleep medication, benzodiazepines and Z-drugs, for adolescents and young adults has decreased in the period of 2015–2018 and is used mostly for 18–24-year-olds [25]. However, unconventional sleep medication, including melatonin, promethazine and low-dose quetiapine, has increased for both age groups [25,26].

Danish guidelines on sleep medication differ for different age groups and medicaments. National guidelines for melatonin targets children and young adults aged 5–20 [21], children aged 6–18 with ADHD [22] or aged 2–17 with autism [23]. Melatonin is approved for adults >55 years of age for jetlag as well as children aged 2–18 years with autism or aged 6–17 years with ADHD [27]. All other prescriptions are off-label, and furthermore long-term effects of melatonin in children are still not sufficiently explored [21].

Prescription of melatonin is recommended to be initiated by or in collaboration with specialists in either child and adolescent psychiatry or pediatrics for patients younger than 18 years [21–23]. Treatment length should be as brief as possible, and deprescription of melatonin for the ≤20-year-olds is recommended if there is no to small effect of melatonin on the sleep. In addition, there should be a reassessment of the indication every six months with a 14-day break [21–23]. Benzodiazepines and Z-drugs are recommended for a maximum of 1–2 weeks and only in cases of acute insomnia [28]. The antipsychotic drug quetiapine, though off-label for sleep issues, is reported to be increasingly used at a low dose for sleep [29] and can be used in special cases where there is psychiatric comorbidity [24].

A relatively small number of studies have been made on the topic of medical practitioners’ reflections when prescribing and deprescribing sleep medication for adolescents and young adults [30]. The deprescription studies focus mostly on older populations [31,32], on deprescription of psychotropic medication in general [33,34], or on conventional sleep medication such as benzodiazepines [31,35,36]. Studies on the prescription of sleep medication, though more researched, are based mostly on quantitative surveys [37–39], thus not focusing on the reflections on prescription habits.

### Aim

This study aims to explore general practitioners’ (from now on GPs) and child and adolescent psychiatrists’ (from now on psychiatrists) considerations when prescribing and deprescribing sleep medication to adolescents and young adults.

## Methods

### Study design

Since the aim of this study was to explore the medical practitioners’ considerations regarding sleep medication, a qualitative interview study was chosen. Qualitative interviews are a useful way to explore the motives and intentions behind the habits of the practitioners, thus aiming to provide a more nuanced and comprehensive understanding of the medical practices of the different practitioners [40]. Therefore, individual, semi-structured interviews with medical practitioners from the two medical fields were conducted to further explore this aim.

### Sampling and recruitment

A primarily purposive sampling strategy was used [41], with strategical inclusion of participants to obtain a diverse group on parameters such as geography, years of practice, age and sex (see Table 1). For example, inclusion of female GPs ceased when an appropriate number was reached, and instead only males were included from that point on. Potential participants were contacted through email with information on the project. In total, 17 GPs and 16 psychiatrists were approached for this study, with 16 replying in due time to be included. Of the 16 participants 10 were GPs, and six were psychiatrists or in the late stages of their medical specialization training. Contact information of the GPs was retrieved through the database medcom.dk. The psychiatrists were contacted through various psychiatric out- and inpatient hospital clinics specializing in adolescent psychiatry. This was further expanded to include a privately working psychiatrist through contact of already included participants, thus transitioning into a snowball sampling strategy [42]. To achieve high information power in our sample, inclusion of participants was continued until we reached data saturation [43].

**Table 1.** Characteristics of participants.

	General practitioners (no. 10)	Child and adolescent psychiatrists (no. 6)
Sex		
Female	6	2
Male	4	4
Mean age in years (range)	50 (42–67)	45 (36–59)
Mean years of practice (range)	13 (4–27)	11 (3.5–26)
Region		
Capital	5	3
Zealand	5	2
Southern Denmark		1
Employment		
Self-employed	10	1
Government employee	0	5

### Data collection

For the semi-structured interviews, two interview guides were developed, one for GPs and one for psychiatrists. They were revised by the authors with suggestions for changes being combined. Two pilot interviews were conducted with a GP and a psychiatrist, respectively, and the interview guides were then further revised. The final interview guides can be found in the appendix as supplementary files A and B. The focus of the interviews was the participants’ considerations when prescribing sleep medication for adolescents and young adults respectively as well as their thoughts on and experience with deprescription: when to initiate it and when to continue the prescription.

The interviews took place between August and November 2023, either at the participants’ workplace or in their private home, and were audio taped and transcribed verbatim. Two interviews were conducted online due to logistical issues. All interviews were conducted in private by NMA, MD, under guidance of supervisor GO, who has many years of experience with qualitative interviews and guidance of these. The privately employed participants were offered a reimbursement for the time spent on the interviews.

### Ethical considerations

All included participants in this study are medical professionals. Participants were provided with clear information about the purpose of the study, and all gave written consent to participate and for the interviews to be used for this study. We ensured that the information about all the participants was anonymized.

### Data analysis

**Figure 1.**
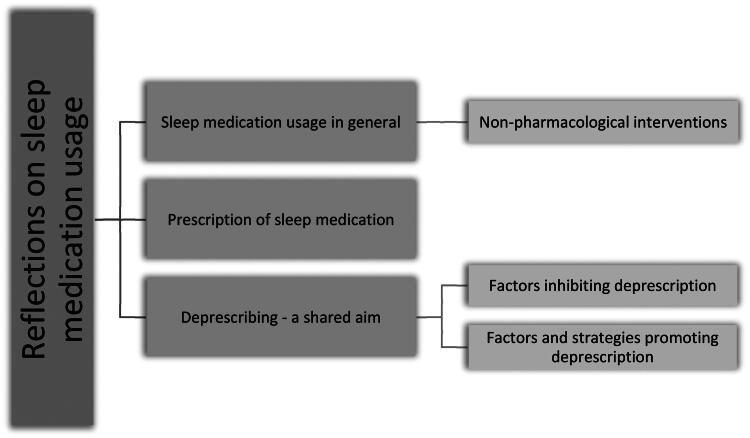
Final thematic code overview.

The interviews were transcribed and coded by author NMA. The transcripts were reviewed and coded using the coding program NVivo (vers. 14). The transcripts were coded using an inductive approach, as to best ascertain reoccurring concepts in the interviews [44]. These concepts were then combined into potential themes and subthemes after a discussion between NMA and GO. They can be seen as supplementary file C. These were then further revised by rereading the different extracts to judge whether a coherent theme ensued, as well as, if these showed an accurate representation of the entirety of the interviews [45]. This resulted in the final thematic template with themes and subthemes as shown in Figure 1. In reporting, the COREQ checklist was used in order to promote a comprehensive and transparent reporting [46]. It is available as supplementary file D.

## Results

The participants’ characteristics are listed in Table 1. Of the psychiatrists, four were working in outpatient clinics, one at a psychiatric inpatient hospital, and one was a privately working psychiatrist. All the GPs worked in their own clinic.

The interviews lasted between 44 and 74 min, averaging about one hour.

The interviews revealed three major themes, with an overall consistency between the participants. These themes are: sleep issues in adolescents and young adults in general, prescription of sleep medication, and deprescription – a shared aim.

### Sleep issues in adolescents and young adults in general

Most GPs mentioned that sleep medication usage in the 13–18-year-olds and 18–24-year-olds was not a very prevalent problem in their practice, with only few patients in these age groups using sleep medication. The adolescents and young adults with longer prescriptions were mostly former patients in the child and adolescent psychiatry and most often diagnosed with ADHD or autism.

However, more than half of the GPs experienced an increase in the number of adolescents and young adults complaining of sleep problems over the last few years, often in connection with generally worsened mental health, as one GP noted:

Sleep medication takes up a lot of time in general practice, and children’s well-being takes up a lot of time in general practice, especially after COVID. Honestly, it’s become more and more, I was about to say worse and worse. And … it’s a huge, huge challenge for us. Also, [there are] more and more medicated children and that is of course for various diagnoses, but also in relation to sleep disorders, yes. (Person 2, GP)

Some GPs mentioned patients directly seeking a prescription of sleep medication, most often the 18–24-year-olds. For the 13–17-year-olds, some GPs experienced parents as the main advocator for pharmacological treatment of their children. The request would often be for melatonin, and patients and parents would be more acquainted with the medicament than previously experienced. Many related this to melatonin becoming more well-known in society, and one GP described his patients’ thoughts as ‘it’s almost like it isn’t medication. Actually, it’s almost healthy. So that, it’s not such a taboo to be asking for it’ (Person 12, GP). This attitude was similarly observed in the child and adolescent psychiatry.

All psychiatrists perceived sleep disorders as a very prevalent problem among their patients, with most experiencing an increase over the years. Regarding the demand for sleep medication, one psychiatrist noted: ‘It’s massive. A massive demand’ (Person 13, psychiatrist). Many psychiatrists explained that they saw more patients with sleep disorders as a result of larger intake of patients in the clinics, a generally worsened mental health in the group, as well as an increase in ADHD and autism diagnoses.

#### Non-pharmacological interventions

Non-pharmacological interventions such as giving advice on sleep hygiene, weighted products, limiting screen time, mindfulness and meditation were unanimously mentioned by all participants as their primary step in solving sleep issues in their patients. Some recommended therapy either at the practitioner themselves, or, if the patients had private means for it, at a psychologist.

### Prescription of sleep medication

GPs generally aimed to avoid prescribing any kind of sleep medication, often relating this practice to current guidelines. Some found that prescription of sleep medication was included in the guidelines advising against prescribing psychotropic medication in general practice for patients under the age of 25, others explained melatonin usage as off-label outside of ADHD and autism treatment. In both cases, these GPs would refer the patient to a psychiatrist if they deemed sleep medication necessary. Additionally, most GPs had an overall aversion toward sleep medication caused by the possibility of side effects, including addiction, as well as a skepticism of the actual effect of sleep medication on the sleep issue, with many noting that it would not address the underlying root cause.

However, in a few circumstances some GPs found it necessary to prescribe sleep medication. This could be when the young person is in acute distress, such as when experiencing sudden death in the family or other acutely traumatic experiences, or in the initial phase of treatment with antidepressant with sleep issues as a comorbidity. In these instances, short-lived benzodiazepines or Z-drugs for a very short period were most often mentioned. The sedating antihistamine promethazine was also mentioned, but large package sizing and a ‘hang-over’ side effect the next day would often deter GPs from using it. A few mentioned recommending herbal medicaments, such as valerian, to their patients.

For melatonin, the GPs were more divided as to prescribing it. Some rarely prescribed it to their patients. One GP explained that the reasoning behind choosing Z-drugs over melatonin was due to the fact that melatonin ‘is not so … present in my mind’ (Person 12, GP). Other reasons mentioned for this was lack of evidence of the effect, worries for the long-term side effects, lack of practitioner’s own knowledge of or experience with the drug, and that it could be considered off-label use. Some would consider represcribing melatonin following a former psychiatric contact if the patient’s conditions worsened.

Other GPs found melatonin useful. This was mostly those who had former pharmacological or psychiatric experience with melatonin or had a patient demographic that provided much experience with psychotropic medication. One GP reflected on their use of melatonin:

I read that the Institute for Rational Pharmacotherapy, they had one (guideline edit.) about melatonin for children. It said that it must be used with care and caution. But… but there’s just not much else to use, I think. I didn’t want to prescribe benzodiazepines or mirtazapine or anything like that. …. I think when the weight of the sleep disorder becomes large enough, I feel that it’s a perfectly reasonable medication to try. (Person 1, GP)

Instances where melatonin was prescribed by the GPs could be in the waiting period before entry into child and adolescent psychiatry, which could last from several months up to a year. This was done to prevent further deterioration of the patient’s condition, and with the expectation that the psychiatrist would be able to reassess the prescription. For some, the prescription would happen after consulting a psychiatrist.

With the psychiatrists, melatonin was mentioned as the primary pharmacological choice, with an overall expression of positive attitude toward the medicament. One participant stated:

I’m of that opinion that it is a substance that the brain itself produces. And I have yet to meet a child who’s experienced side effects … So I’m generous with it. (Person 5, psychiatrist)

All psychiatrists but one mentioned that they tended to prescribe melatonin relatively quickly. Several causes for this were mentioned: that sleep issues have a heavy negative impact on both the child and the family, that the child was not able to attend school, and the apparent fact that children arriving in the psychiatry have consulted a doctor before, yet still have an ongoing problem. As one put it:

I feel that those who come to me, they’ve usually been at someone else before, so when they finally come to me, it’s very easily prescribed. (Person 9, psychiatrist)

In addition to melatonin, low-dose quetiapine was also used in the psychiatry. Opinions on quetiapine were divided, with some considering it highly beneficial, while others found the use ‘more out of necessity than of desire’ (Person 11, psychiatrist). Overall, it was only used in special cases such as severe autism, patients in acute distress or with excessive thoughts. One psychiatrist stated that low-dose quetiapine sometimes could be a substitute for benzodiazepines as they experienced that side effects were less common. The possibility of side effects of the antipsychotic weighed heavy on every psychiatrist, who would monitor closely for these. Some attributed the increased usage to a newly recognized effectiveness of low-dose quetiapine, particularly in managing comorbidities in autism.

Two GPs, whose uptake area included a patient demographic with social problems, used low-dose quetiapine for a short period in incidents when patients experienced sleep problems linked to withdrawal symptoms from drug abuse or experiencing psychosis-like symptoms. Other GPs mentioned only represcribing quetiapine following a former psychiatric contact.

There seemed to be a disparity among the two groups of practitioners when it comes to the indication for sleep medication and the severity of sleep issues. Several of the GPs found sleep issues to be less of a concern, stating, for instance, ‘Well, a lack of sleep won’t kill you’ (Person 6, GP) and ‘I tell them (patients edit.) that sleep issues are never dangerous’ (Person 14, GP). Most psychiatrists on the other hand considered lack of sleep rather serious: ‘But I actually see lack of sleep as something pretty critical’ (Person 5, psychiatrist) and would more often opt to treat the condition pharmacologically to help the patient on the matter.

### Deprescription – a shared aim

Every participant expressed a desire to deprescribe their patients’ sleep medication. For melatonin, psychiatrists would usually try to follow the guidelines with a biannual pause for 14 days to decide whether the melatonin was still indicated, which it generally would be. Many psychiatrists acknowledged the guidelines recommending as short a treatment length as possible. However, most expected the prescription to last for several years or as long as the patient would have substantial sleep issues. Therefore, most agreed that the deprescription of melatonin would usually take place in general practice and very few psychiatrists had experienced deprescribing melatonin themselves. As one psychiatrist put it:

We tell the family and the young person that it is a short-term treatment. To reduce sleep latency in a vulnerable period of the child’s life or the young person’s life, and that, that it’s not something which is lifelong … I most often experience that it doesn’t really work like that, or that it’s not realistic because many of our young continue the treatment and are passed on to general practice with their melatonin … So it’s, I think that I believe that it’s a treatment that’ll continue into adulthood. (Person 16, psychiatrist)

The psychiatrists generally expressed a belief in the GPs’ abilities to monitor and deprescribe melatonin when time comes. However, one psychiatrist noted an uncertainty as to how the prescription would continue in general practice:

It is my impression that they’re not that used to using the medication (melatonin edit). Because of that, I can be a bit uncertain as to whether they have the opportunity to do the actual work and have them take a break for 2 weeks and then do a reassessment … I actually have no idea how they actually do it. It’s also bad that I don’t know. But I actually don’t know … how it is handled in general practice when they (patients edit) bring treatment with melatonin with them from here. (Person 7, psychiatrist)

Psychiatrists would also experience different responses from the GPs when discharging a patient medicated with melatonin to general practice, with some accepting it without issue or asking for more guidance and others declining it. It was not a general habit for most of the psychiatrists to hand over specific guidelines or a plan to the GPs on how to monitor or deprescribe melatonin. In terms of low-dose quetiapine, however, most mentioned it as being important to deprescribe before turning the patients over to GPs or recommend a referral to an adult psychiatrist to continue the prescription.

For GPs, deprescription was divided into two: deprescription of prescriptions initiated by themselves and those initiated in the psychiatry. For their own prescription, the GPs had a very firm idea of when to deprescribe. They would usually advise the patient from the onset that the prescription was intended for a limited period and keep to this.

With the prescriptions made in the psychiatry, usually melatonin, the approach differed. After reflecting a bit upon their practice and comparing prescription made in the psychiatry or on their own, one GP noted:

Yes, it’s a bit funny, because I think I’m probably much better at it (deprescription edit), when it’s me who initiated it. I do think sometimes it’s hard, you know, when it’s another person who initiated it (the prescription edit)… But it’s probably because you think: ‘Well, they have been to a specialist’. It can be a child and adolescent psychiatrist, where you think: ‘Ah okay, but there’s probably some reason or another for this being prescribed’. And then I’m perhaps a bit more restrictive about taking them out of it again. (Person 4, GP)

This sentiment was repeated by several of the other GPs, with some seeming surprised by this realization of the difference. When taking over the prescription made by psychiatrists, some GPs would call the patient in for an evaluating consultation on the treatment they have had, where a few would consider discussing deprescription with the patient. However, most would wait, deeming the patient too vulnerable to be begin deprescription or perceiving that they would meet a considerable opposition from the patient and parents at this early stage.

Deprescription conversations might then take place later on. Most would have an annual conversation with the patient on their prescriptions with the opportunity to bring up the possibility of deprescribing. However, many GPs mentioned that deprescription was rarely brought up in practice in these routine checkups. Some would see them every three months or with every renewal of the prescription, and some would not have any standard routines. Only one GP mentioned using a 14-day to a month-long break yearly to judge whether the prescription was still needed.

#### Factors inhibiting deprescription

GPs mentioned that occasionally it could be quite easy to overlook the prescriptions made in the child and adolescent psychiatry. As one put it: ‘Someone smart has decided they needed it’ (Person 12, GP). Therefore, some found they did not automatically consider if the prescription was necessary to continue and when to consider deprescribing. Additionally, having a busy schedule with many patients throughout the day would often make it harder to do more specialized medical work and keep a focus on this: ‘It takes time. It takes energy. To talk. Motivate all the time. Motivational conversations take time’ (Person 3, GP). Instead, many found themselves just doing ‘the easiest thing’ (Person 2) and continuing the prescription.

Another inhibiting factor for the GPs was the possibility of a long wait before the patient gained access to a psychiatrist after a referral in case the patient’s state worsened after the deprescription. As one GP put it:

I think some of the uncertainty also comes from the fact that I know I have to wait a year to get them into the psychiatry… therefore, I also become more careful with what I do, because I know that if it goes wrong, then the patient is worse off, because it’s difficult to get a new appointment… if it suddenly turns out that something else is needed, then I feel that it can be really difficult to get any help. (Person 15, GP)

Similarly, both GPs and psychiatrists mentioned the fear of destabilizing their patient by deprescribing too early. Both acknowledged that patients would often be in a very vulnerable state at this age, emphasizing that an untimely deprescription could negatively impact both academic performance and future prospects.

Lastly, several GPs and a few psychiatrists mentioned that a pressure from patients or parents could be a contributing factor to continue prescribing. Many practitioners would agree to this due to the belief that melatonin carries few side effects. This was a consensus many reflected upon, concluding that the lack of severe adverse reactions made it less crucial for them to deprescribe: ‘Again, then you shrug and think; no harm, no foul’ (Person 11, psychiatrist).

#### Factors and strategies promoting deprescription

To help deprescribing, several participants mentioned having very conscious focus on not making patients ‘chronic’ (Person 2, GP) and thus avoiding facilitating a prescription habit for the patient to continue. One approach was to inform patients that the prescription was not to be considered lifelong, aiming for deprescription when possible. This was generally well received by patients and parents and made deprescription more likely to be successful.

Some GPs noticed that former experience with deprescription of adult benzodiazepine use was contributing with a focus on deprescription in this age group:

since I was so focused on benzo deprescription to begin with, I wanted to, I wished to do that for children and young people as well. (Person 3, GP)

Furthermore, it was mentioned several times that deprescription of melatonin often depended on the patient and their motivation. Consequently, in many cases, the deprescription would happen on the patient’s own initiative:

Typically, it will also be that the child has become more mature or something. Has gotten other… strategies that make it (sleep medication edit.) unnecessary. That will typically be what they come up with… so they have also thought about it themselves. (Person 8, GP)

Many practitioners mentioned that the young patients usually had an aversion to taking medication, making young patients more motivated for deprescribing. Besides, some recalled patients who had forgotten to take their medication and discovered they could sleep without it, leading to deprescription.

Regarding potential promoting factors, some GPs found that more clear guidelines, as well as guidelines aimed at primary practice, would help to improve the deprescription of melatonin: ‘I don’t think there’s these very clear guidelines, like: “Do it like this”. There’s not this clear recipe’ (Person 4, GP). Moreover, some GPs expressed that a plan in the letter of discharge from the child and adolescent psychiatry would help deprescription. One GP mentioned that it would give ‘… more control with it. As it is now it isn’t well-managed. As it is now it’s disorganized’ (Person 12, GP).

One GP found that the current system and GPs in general lacked focus on deprescription of sleep medication and suggested that further education on monitoring and deprescribing sleep medication would help improve this:

I don’t feel like therés focus on this (sleep medication, edit) … Also, in the continuing training there isn’t enough focus on it, even when we’re talking about sleep … It’s adults… But not children. It’s rarely children and young people … I think we need to be more conscious that child and adolescent psychiatrists are not going to monitor them until they’re tapered (in sleep medication edit). They don’t do it. We need to do it. So we need to be more conscious of this. (Person 3, GP)

## Discussion

Findings on practitioners’ considerations when using sleep medication for the 13–24-year-olds suggest that GPs had varying familiarity with prescribing sleep medication for these age groups. However, many participants had experienced an increase in contacts regarding sleep issues. Psychiatrists typically prescribed melatonin expecting it to last for years and be deprescribed in general practice. GPs found it easier to deprescribe their own prescriptions than those made by psychiatrists. Other factors inhibiting deprescription were concerns over destabilizing the patient, the long wait before patient entry to the psychiatry, time constraints and potential resistance from patients and their family. Participants found that guidelines aimed at general practice or recommendations for deprescribing presented in the letter of discharge would make deprescribing easier, combined with a motivated patient and having conscious focus on deprescription.

A relatively small number of studies have been made on the topic of medical practitioners’ reflections when prescribing and deprescribing sleep medication for adolescents and young adults [30]. The deprescription studies focus mostly on older populations [31,32], on deprescription of psychotropic medication in general [33,34], or on conventional sleep medication such as benzodiazepines [31,35,36]. Studies on the prescription of sleep medication, though more researched, are based mostly on quantitative surveys [37–39], thus not focusing on the reflections on prescription habits.

Extensive research exists on deprescribing sleep medication such as benzodiazepines and Z-drugs in older adult populations [47–49]. Additionally, some studies have explored the clinical practices in prescribing and deprescribing different psychotropic medicaments in children and adolescents [34,50,51]. However, our study fills a critical gap in the literature by exploring the challenges of deprescribing sleep medication, including melatonin, in the 13–24-year-old age group. This population is often caught in a transitional phase between pediatric and adult care, where existing guidelines may not fully apply. Our findings correlate well with the current prescription data [25], with melatonin being the most commonly prescribed medication and to a lesser degree benzodiazepines and Z-drugs, promethazine, and low-dose quetiapine. The prescription habits of the practitioners also reflect the current guidelines [21] since non-pharmacological interventions were used as first choice treatment, and sleep medication mostly being prescribed by psychiatrists.

The use of low-dose quetiapine was similar to what other qualitative studies on quetiapine use have discovered, finding it prescribed for more complicated psychiatric diagnoses and occasionally instead of benzodiazepines [30]. The psychiatrists and GPs who prescribed quetiapine in this study would, however, monitor and be very focused on the metabolic adverse effects compared with the practitioners in the referenced study.

Many of the inhibiting factors for deprescribing sleep medication uncovered in this study is similar to existing literature on the topic [35,52]. Another study also found a difference in attitude toward discontinuing a prescription made by another practitioner compared to one made by the medical practitioner themselves [30]. Similarly to our study, Kelly et al. discovered that practitioners found it more difficult to deprescribe when they did not know the reason behind the prescription. Furthermore, this study found that barriers such as parental opposition made it harder for practitioners to deprescribe. In addition to this, the fear of destabilizing a patient and the lack of confidence in one’s ability to handle it can also be found in other literature as a barrier to deprescribing in general in general practice [52].

This study found that deprescribing sustained a big workload on GPs, and the extra time it ensued would keep them from deprescribing. This has been found in other studies as well: a crowded health system with limited resources makes it hard to find the time for follow-up on a deprescription [34]. Correspondingly, this study found that lack of guidelines and education caused a reduced focus on the area and uncertainty among GPs, making it easier to just continue the prescription. This has also been found in prior literature on deprescribing [52] and in former qualitative studies in a general practice setting on older populations [32].

### Strengths and limitations

The study encompasses several strengths. First, a rich and comprehensive sampling of participants in terms of age and sociodemographics was achieved. Furthermore, the use of qualitative semi-structured interviews gave a thorough understanding of GPs’ considerations when prescribing and deprescribing sleep medication for 13–24 year-olds. The interview guides were meticulous and were adjusted after pilot interviews [41]. The length of the interviews, averaging around one hour, gave room for deep reflections for the participants as well. The COREQ guidelines were followed in order to provide transparency in our reporting [46], and themes were discussed between the authors [45].

During the interviews, it became apparent that practitioners’ management of sleep medication is a potential sensitive subject, especially for GPs. It took some time for many of them to recall and reflect upon the prescription of sleep medication in their practice. This further implicated the usefulness of the semi-qualitative interview method since it encouraged the participants to reflect upon their practices. However, the sensitivity of the topic may have produced more closed answers.

Furthermore, the sample of participants would ideally have included more privately working psychiatrists as they in particular prescribe melatonin to the under 18-year-olds, as mentioned and experienced by several of the GPs.

### Implications for practice, policies, training and research

This study provides several possible implications for clinical practice, health policies, medical training and research. The findings indicate an inconsistency in GPs’ deprescription practice of melatonin caused by several complicating factors. This suggests a need to promote discussion within general practice on the topic of sleep medication for adolescents and young adults as well as considering expanding GPs’ continuing education on the area.

Moreover, there seemed to be an expectation of the psychiatrists that the prescription of melatonin would last into the patients 20s with deprescription taking place in general practice. However, current guidelines on melatonin only target people below 21 years and above 55 years. This study therefore encourages authorities to consider expanding the targeted age for guidelines on melatonin, including persons with and without psychiatric comorbidities. Additionally, psychiatrists did not typically provide GPs with a plan of deprescription. Concurringly, several GPs expressed a wish for a plan in the letter of discharge to help future deprescription. Improved cross-sectional communication between the two medical fields could therefore prove beneficial.

Due to the sensitive nature of this subject for the participants, it would be recommendable to further expand the research on this topic by examining register-based data on prescriptions as well as video observations in private practice. This could likely lead to a deeper understanding of what the actual clinical practice involving prescription and deprescription looks like [53–55].

## Conclusions

Prescription of unconventional and off-label sleep medication has been increasing for adolescents and young adults in recent years. This study provides a unique insight into medical practitioners’ considerations when using sleep medication for these age groups. Findings suggest that medical practitioners experience an increase in contacts regarding sleep issues among young people. When prescribing melatonin to adolescents, psychiatrists expected it to last years and be deprescribed in general practice. However, there seemed to be a discrepancy as to how the deprescription should unfold among the practitioners, as well as several factors complicating the deprescription in general practice. This suggests a need for considering expanding guidelines, improving cross-sectional communication between general practice and child and adolescent psychiatry, as well as providing further continuing education for GPs on this area.

## Supplementary Material

Supplemental Material
