# Supplementary material for: Medical practitioners’ experiences and considerations when managing sleep medication for adolescents and young adults: A qualitative study on general practitioners’ and child and adolescent psychiatrists’ reflections on their prescription and deprescription practices of sleep medication for 13–24-year-olds
Source: Scand J Prim Health Care. 2024 Sep 30;43(1):120–30. doi: 10.1080/02813432.2024.2407877 (PMC11834797; doi:10.1080/02813432.2024.2407877)

**SUPPLEMENTARY FILES**

*Supplementary file A*

**Interview guide: Child and Adolescent Psychiatrists**

Purpose of the interview: To examine the considerations as a psychiatrist regarding prescription of sleep medication in adolescents (aged 13-17).

Particularly regarding prescribing and expectations regarding deprescription of the prescribed medication later on.

**About the interviewee**

Name, age, briefly about level of education and experience within their field. Experience within other fields.

In brief terms, describe your workplace and working procedures?

Who attends the patients?

How often do you encounter the group in question?

**Initiation/prescription**

Begin with an example of a patient with sleep problems to whom you prescribed sleep medication (a patient with ADHD, OCD, eating disorder, depression, autism – preferably not bipolar or psychotic).

How was the patient met?

What do they complain about?

Define “sleep problems”?

What made you initiate treatment with sleep medication?

Thoughts about the prescription?

An urgent need?

What is the role of parents? (Pressure from patient/parents regarding treatment of the sleep problem)

Patient/parents’ expectations regarding sleep?

What thoughts do they have about “good sleep”?

What complaints do they have/what goal?

Describe a usual plan for/approach to a patient with sleep problems?

E.g. a weighted blanket

Improvements to sleep hygiene

CBT

Difference in clinical use of melatonin, quetiapine, mirtazapine, sedating antihistamines and, if relevant, benzodiazepines/Z-drugs?

What are your thoughts about this?

What is your experience with long-term treatment with low-dose quetiapine?

When prescribing sleep medication, do you at this point make any considerations regarding deprescription?

Do you discuss deprescribing with your patients at the initiation?

What is your experience with sleep medication in different age groups?

Please elaborate on your thought process regarding prescription of sleep medication?

When do you prescribe, and when do you not?

Cost/benefit?

In this regard, lack of knowledge about long-term side effects.

**Continuation/monitoring**

Who does follow-ups on patients after initiating treatment?

Who is monitoring the patient? Nurse? What are they supposed to pay attention to?

How does the monitoring work?

Considerations regarding duration of treatment.

What do you expect will happen to the prescription after you have initiated treatment with it?
If patients are discharged with prescribed sleep medication?

Is a limit set to the number of times the medicine can be handed out?

Do you expect the general practitioners to handle the continuation of the prescription?

**Deprescription**

Try to describe one of your patients’ course of deprescription?

Imagine you have a patient whose disorder has bettered. She wants to sleep without use of medication. How do you make a plan? What alternatives do you find?

Talk about the barriers preventing deprescription?

Patient/parents?

Lack of guidelines?

Are there any finds that make you deprescribe/taper a prescription?

Improvement of sleep?

Would you consider deprescribing the prescription when the patients’ sleep pattern has returned to normal?

Lack of effect on sleep after prescribing sleep medication?

Attempting alternative ways to improve sleep?

If you discover a prescription with long duration which was only supposed to last for a short time?

**Opinions**

Considerations regarding deprescribing

Who should initiate the deprescription?

Who has the responsibility?

General practitioners?

Are there any conversations in the workplace regarding deprescribing and prescription of sleep medication?

A conversation between colleagues about it?

Does everyone approach the problem the same way?

Have you experienced a development in the use of sleep medication over time? According to you, what is the reason for the increase that has happened?

Regarding use of low-dose quetiapine and melatonin.

Have you experienced increase in the demand for sleep medication?

The development of bad sleep?

Complaints about bad sleep?

According to you, is too much sleep medication being prescribed in general?

The consequences of this.

According to you, are there any options to lower the consumption of it?

Alternative measures before prescription of medication

Education of patient/parents

Guidelines

CBT

*Supplementary file B*

**Interview guide: General Practitioners**

Purpose of the interview: To examine the considerations of general practitioners regarding the prescription and deprescription of sleep medicine in adolescents and young adults (aged 13-24).

Sleep medication defined as either melatonin, low-dose quetiapine, sedating antihistamines, benzodiazepines and Z-drugs, mirtazapine or other.

**About the interviewee**

Name, age, briefly about level of education and experience within their field.

In brief terms, describe your workplace and working procedures?

How often do you encounter the age group in question?

How are they usually distributed between ages 13-24 years?

**Initiation/prescription**

Begin with an example of a patient

Characterize the sleep problems?

Have you initiated treatment with sleep medication in young patients? If so, with which medicament?

What made you initiate treatment with sleep medication? Thoughts about the prescription?

An urgent need?

Pressure from patient?

(What was the role of the parents?)

Request?

What considerations did you make making the prescription?

Regarding pros/cons

Regarding background for the sleep disorder

Substance abuse?

When prescribing sleep medication, do you at this point make any considerations regarding deprescription?

Do you discuss deprescribing with your patients at the initiation?

What about a patient aged 13-17 (or 18-24)?

Is there a difference between the two groups?

How do you usually handle sleep problems in young patients?

Do you delegate (some of) the tasks to other professionals in the clinic?

Describe a usual plan for/approach to a patient with sleep problems?

E.g. a weighted blanket

Improvements to sleep hygiene

CBT

Referral?

In which situations do you make a referral to a specialist instead of initiating treatment yourself/or the other way around?

What is your experience with the use of melatonin, quetiapine, sedating antihistamines and benzodiazepines or other sleep medications, respectively?

What are your thoughts about this?

What is your experience with low-dose quetiapine as a “sleep medication”?

**Continuation/motitoring**

How do you follow patients after initiating treatment with the prescription?

Who is monitoring the patient? Nurse? What are they supposed to pay attention to?

What about guidelines?

Considerations regarding duration of treatment.

What are your thoughts regarding maintaining the prescription?

What makes you continue the prescription?

What is your experience with those patients (in the age group in question) who are discharged with sleep medication from the psychiatric sector?

Do you have an example of a patient who was discharged from the psychiatric sector with sleep medication to be followed up on with their GP (you)?

What was the course of treatment like?

What considerations did you make along the way?

How did you follow up on them?

**Deprescription**

Try to describe one of your patients’ course of deprescription?

Imagine you have a patient who has been prescribed a type of sleep medication for a relatively long period of time. She wants to sleep without medication. How do you make a plan? What alternatives do you find? Do they work?

Are there any barriers that prevent deprescription?

Patient-related

Parents?

Belief in the ability to sleep without medication? Patient education?

Lack of alternative treatment

Professional-related

Lack of guidelines?

Lack of professional discussion with colleagues/the psychiatric sector

Lack of experience with psychiatric medication

Unsureness related to deprescription. Are you comfortable with it?

Are there any finds that make you that makes you deprescribe/taper a prescription?

Improvement of sleep?

Would you consider deprescribing when the patients’ sleep pattern has returned to normal?

Lack of effect on sleep after prescribing sleep medication?

Attempting alternative ways to improve sleep?

If you discover a prescription with long duration which was only supposed to last for a short time?

Lack of knowledge regarding long-term side effects.

Difference between deprescribing different medicaments

When do you make a referral?

**Opinions**

Are there any conversations in the workplace regarding deprescription?

A conversation between colleagues about it?

Does everyone approach the problem the same way?

How are the options for counseling/cooperating with colleagues in BUP/the region regarding sleep medication?

Regarding initiation/continuation?

Regarding deprescription?

According to you, who ought to be responsible for deprescription?

Difference between different medicaments?

According to you, what could increase deprescription?

Guidelines

Patient education

According to you, how could one become better at deprescription? What changes are needed in order for you to make a decision regarding deprescription in the same way as initiation?

Have you experienced a development in the use of sleep medication over time? According to you, what is the reason for the increase that has happened and still is happening?

Do you think, in general, that too much sleep medication is prescribed?

Have you experienced increase in the demand for sleep medication?

The development of bad sleep.

Complaints about bad sleep?

According to you, are there any options to lower the use of it?

Alternative measures before prescription of medication

Education of patient/parents

CBT

What is your experience with the patient’s/parents’ expectations regarding sleep?

E.g. how to define “good sleep”

*Supplementary file C*


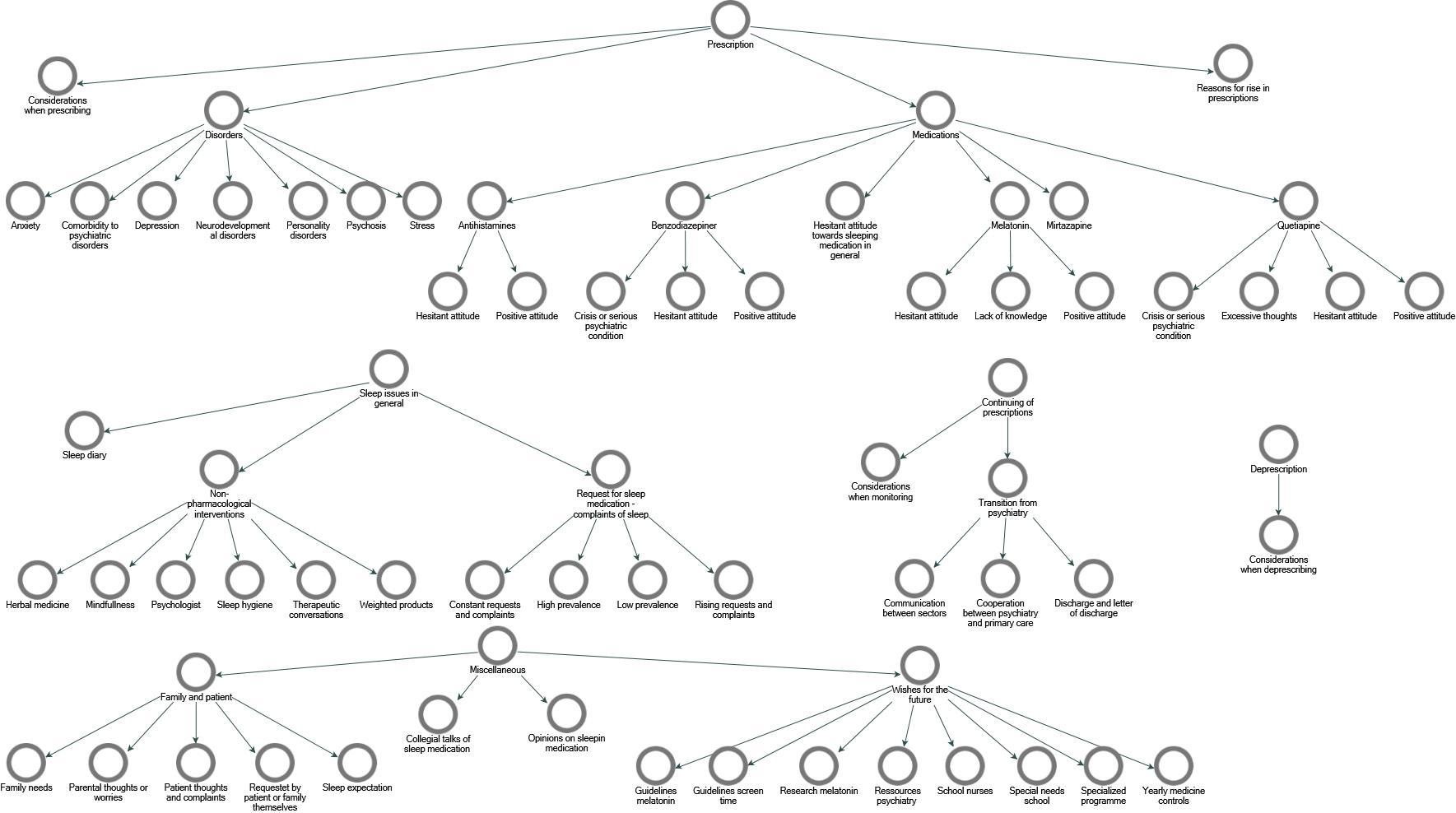


*Supplementary file D*


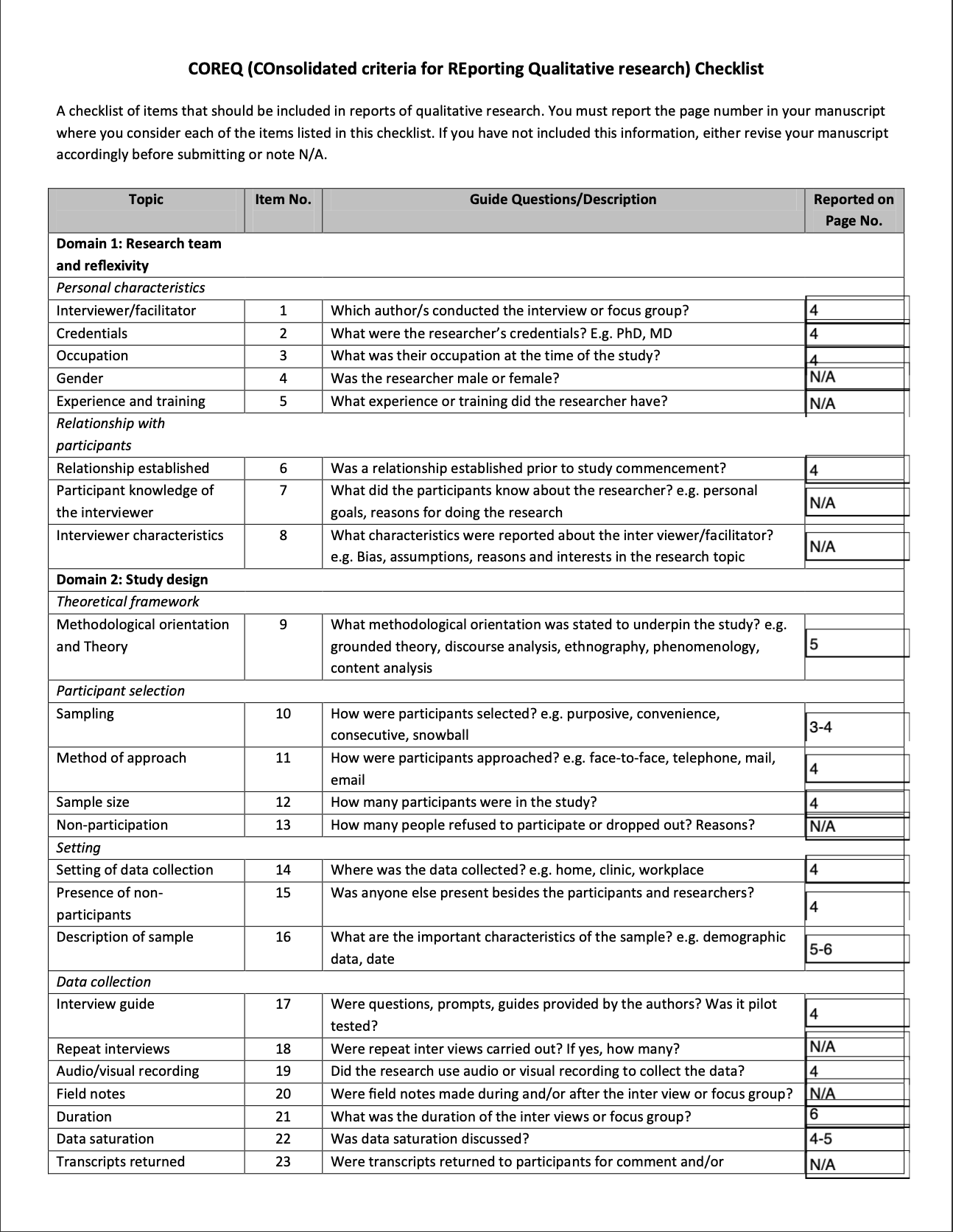


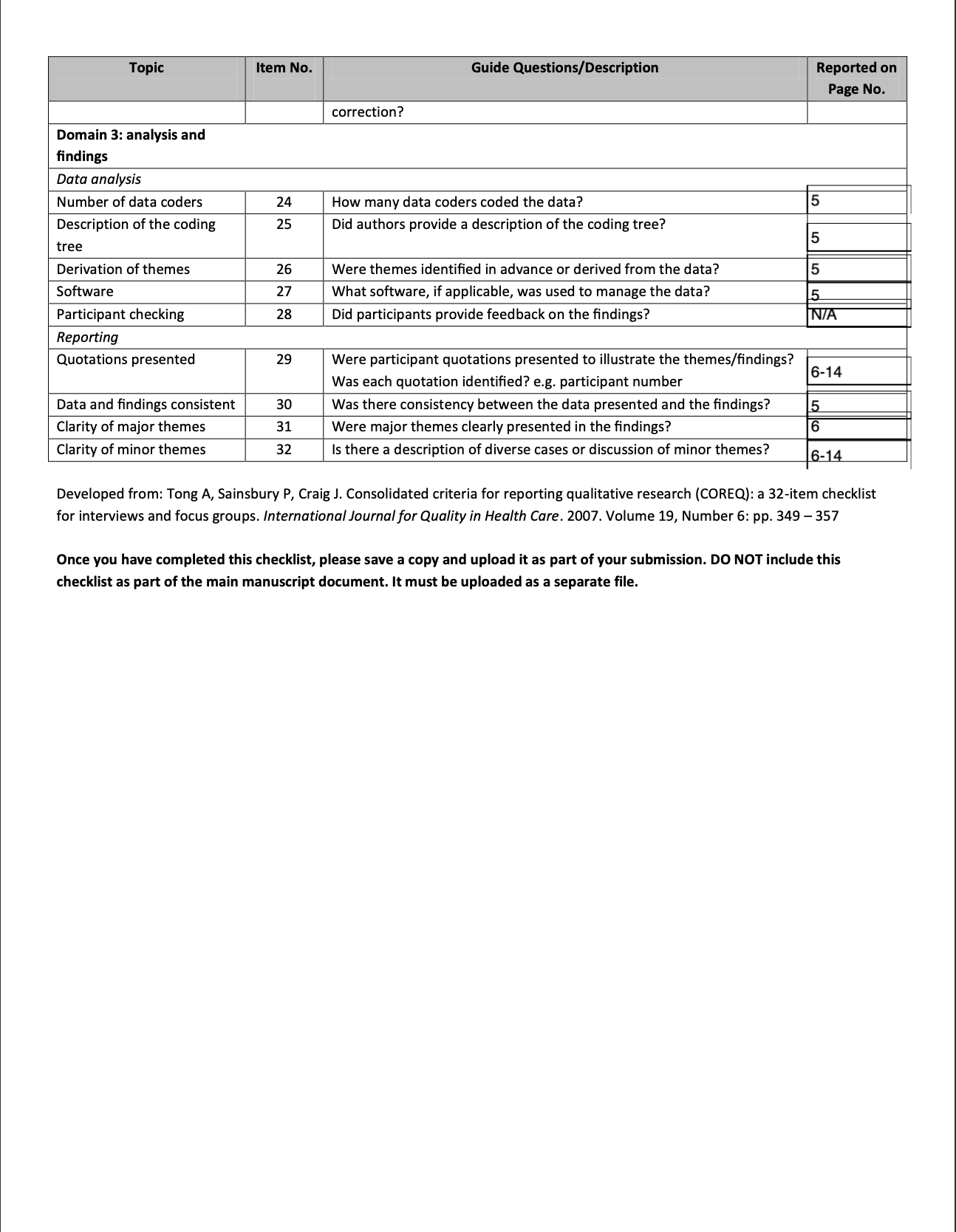

Supplement: Supplemental Material [file IPRI_A_2407877_SM6653.docx]
